# The effects of app-based mindfulness practice on the well-being of university students and staff

**DOI:** 10.1007/s12144-021-01762-z

**Published:** 2021-05-01

**Authors:** Oskari Lahtinen, Jenni Aaltonen, Johanna Kaakinen, Lena Franklin, Jukka Hyönä

**Affiliations:** 1 Department of Psychology and Speech-Language Pathology, University of Turku, FI-20014 Turku, Finland; 2 Atlanta, GA USA

**Keywords:** Mindfulness, Mindfulness-app, Anxiety, Depression, University staff, Students

## Abstract

Mental health problems like anxiety, depression, and stress have been increasing in many countries and the 2020 COVID-19 pandemic has further exacerbated their toll. Mindfulness-based interventions have been shown to provide evidence-based treatments for anxiety and depression, and accumulating evidence is emerging in support of using mindfulness apps yielding small-to-moderate treatment effects. The study was a 4-week randomized controlled trial with 561 university students and staff as participants, divided into a treatment group (mindfulness app) and an active control group (psychoeducational online content). Depression, anxiety, and stress were evaluated as primary study outcomes. Saliva cortisol samples were also collected from a subgroup of the treatment arm (*n* = 29). Using the mindfulness app for four weeks resulted in small reductions in stress (d = .16), and depression (d = .16). Attrition was 28.0%. Subjects who practiced more did not experience additional improvement in wellbeing. Mindfulness apps offer modest but clear benefits to users in terms of improved mental health. They present a promising supplement to traditional mental health services.

Before the COVID-19 pandemic in 2020, around a third of university students reported suffering from mental health problems (Finnish sample; Kunttu et al., 2016). Instead of seeking help, students often self-medicate with alcohol (39%), cigarettes (36%), or drugs (15%; Pierceall & Keim, 2007). At the time of writing, the 2020 COVID-19 pandemic has caused around the world common mental health problems like anxiety, depression, and stress to skyrocket. More than 30% of Americans have reported symptoms of generalized anxiety disorder (peaking in the youngest age group, 18–29-year-olds with 40+ % prevalence). The numbers for depression are nearly as dire with 23.5% for the entire US population and 32.7% for 18–29-year-olds (CDC, 2020). The situation presents a substantial stress test to mental health services around the world. As a result, cost-effective and accessible mental health solutions are in high demand.

Mindfulness meditation has been used to alleviate anxiety, depression, and stress. Meta-analyses support its efficacy for anxiety and depression, while the jury is still partially out on stress (Goleman & Davidson, 2017; Goyal et al., 2014). Strongest evidence is for the efficacy of Mindfulness-Based Cognitive Therapy in reducing the risk of depressive relapse (Kuyken et al., 2015; Teasdale et al., 2000). Another well-researched, and moderately effective, mindfulness-based intervention (MBI) is Mindfulness-Based Stress Reduction (MBSR; de Vibe et al., 2017; Kabat-Zinn, 1990).

Mindfulness meditation is practiced by focusing attention on present moment experience in a nonjudgmental way. Practice usually begins with paying attention to body sensations like the breath. When the mind wanders to thoughts, attention is brought back to the erstwhile focus. Once competence builds, practice can open up to noticing anything in experience and maintaining attention on it. Practice time in hours correlates with how much participants benefit from it (Goleman & Davidson, 2017; Parsons et al., 2017).

With the increased popularity of smartphones, we have experienced a significant upsurge of smartphone applications geared towards enhancing subjective wellbeing and mental health. They are a promising means to enhance human wellbeing, as they are readily available for use by anyone anytime and anywhere. The 2010s also saw a spike in studies examining the effectiveness of smartphone applications in promoting mental health. Unfortunately, however, many of them were found to be ineffective or even harmful. In their recent systematic review, Wang et al. (2018) assessed 100 smartphone applications designed to promote mental health. Only 14 of them were deemed to demonstrate evidence-based effectiveness.

In addition to general mental health apps, the previous decade also saw an explosion in the availability of mindfulness apps in particular. A 2015 survey of mindfulness apps concluded that most did not in fact offer mindfulness and only one out of hundreds was supported by research evidence (Mani et al., 2015). After 2015, studies evaluating apps began to crop up. Mindfulness apps usually offer 10 or 20 min daily guided meditations and these can target specific ailments like stress, anxiety, or depression. Many randomized controlled trials (RCTs) now support the efficacy of Headspace in alleviating, e.g., anxiety, and depression (Bennike et al., 2017; Bostock et al., 2019; Economides et al., 2018; Howells et al., 2016; Noone & Hogan, 2018; Rosen et al., 2018; Yang et al., 2018). Other evidence exists for benefits of, e.g., apps Calm (stress: Huberty et al., 2019; wellbeing: Clarke & Draper, 2020), and Smiling Mind (depressive symptom improvement, resilience; Flett et al., 2019). Apps have been studied in many different populations including physicians (Roy et al., 2020; Wen et al., 2017), academic advisors (Hendricks et al., 2019), and university students (Lyzwinski et al., 2019), with mostly encouraging results. The latter two groups are targets in the present study as well. No meta-analyses have combined effects sizes from mindfulness app studies, though one evaluated online (including website-based) mindfulness-based programs and found salutary main-effects for depression (g = .29), anxiety, (g = .22), and stress (g = .51; Spijkerman et al., 2016).

Mindfulness research has been under criticism for suboptimal methodology of late, and the field has been asked to step up its game (Davidson & Dahl, 2018; Van Dam et al., 2018). Even though several RCTs of mindfulness apps have recently been published, very few studies still use an active control group, the gold standard in MBI research (Goleman & Davidson, 2017). Attrition rates have not been evaluated for mindfulness apps specifically, but a meta-analysis reported a 50% rate for all apps for depressive symptoms collectively (Torous et al., 2020). Sample sizes have also tended to be quite small in studies in the field (most report *n* < 100). An issue in the design of many studies has also been that even though the amount of practice (in minutes) is crucial information, few studies record it. Very few studies have been preregistered to prevent publication bias (Coronado-Montoya et al., 2016). Finally, outcome measures in mindfulness research outside of neuroimaging studies have often tended to be questionnaire-based, which are prone to bias. Biological outcomes would give more accurate information on what is actually happening within practitioners.

The present study is a pre-registered RCT, in which we evaluated a mindfulness app, Welzen, against a specifically tailored active control treatment. The app offered participants guided meditations designed to alleviate symptoms of anxiety, depression, and stress. We recruited a large sample (*n* = 561) from a Finnish university with both students and staff included. A subsample of the treatment arm (*n* = 30) gave salivary cortisol samples pre- and post-study. Our primary hypothesis was that the app would offer small-to-moderate reductions in anxiety and depression, and perhaps in stress, in the treatment arm of the RCT. We also hypothesized that practice minutes would correlate with the amount of benefit participants derive from practice. We expected cortisol levels to decline in saliva samples from pre- to post-measurement. The study was preregistered at Open Science Framework (Hyönä et al., 2018). We collected two waves of data on the RCT design. We also collected exploratory data which are mentioned in the preregistration, but not reported in the study as there was no control group for these data.

## Method

### Participants

**Fig. 1:**
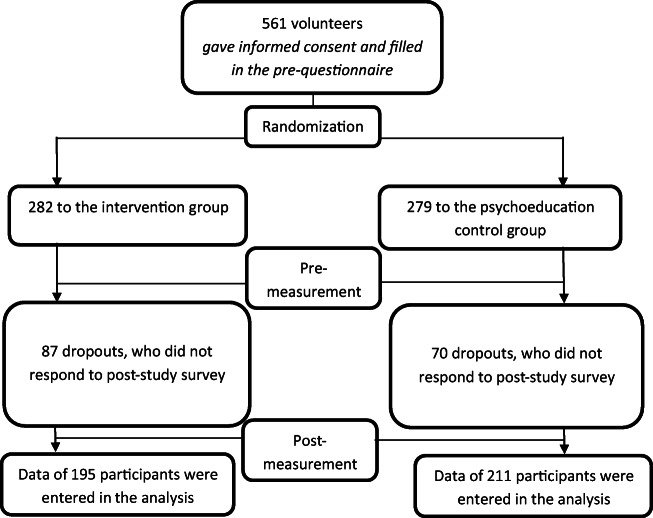
Process chart from recruitment to post-measurement

Participants were recruited from among the faculty, staff, and students of the University of Turku. The University Wellbeing Services helped in the recruitment. The study was advertised via the university email lists, in the intranet, and with fliers distributed in the campus area. Every volunteer was accepted to participate provided they (1) owned a smartphone, (2) had sufficient English skills (the app was in English), (3) had not practiced mindfulness on a regular basis, and (4) was committed to regular practice for four weeks. Volunteers with a psychiatric diagnosis were instructed to consult their therapist/doctor about the suitability for participation. If permitted by the therapist, they were allowed to take part in the study. Thirty-three participants in the intervention group and 32 in the active control group had a psychiatric diagnosis. A total of 561 people volunteered for the study. They were randomly assigned to the intervention and the psychoeducation control group using the randomlists.com website. A graphic depiction of the recruitment process is found in Fig. 1 that also includes the drop-out rate.

### Procedure

The participants volunteered to take part in the study by sending an email to the 2nd author who sent the participant a link to the pre-questionnaires to be filled in. The participants also provided their email address to which a link to the smart phone app could be sent. Moreover, they were asked about their sex, nationality, level and type of education, current academic status (undergraduate or post-graduate student, staff member, faculty member), previous experience with mindfulness meditation, rated interest in mindfulness meditation, and possible diagnosis of a psychiatric disorder and its treatment.

After participants had filled in the pre-study questionnaires, 9 test tubes together with the instructions for taking the samples and a return envelope were mailed to the cortisol group (*n* = 30). They were provided with another 9 test tubes and a return envelope after the intervention was complete.

The members of the intervention group received an email where they were supplied a link to the daily guided meditations. They also received a one-year premium membership for the app, which meant that they had free access to all its meditation practices. The members of the psychoeducation control group were sent a link to the podcasts appearing in SoundCloud they were instructed to listen to (one recording per day, 6 days a week). They were told that after one month they will be given access to all the contents in the app.

All participants received an email on four consecutive Fridays during the intervention that encouraged them to continue with the program. Immediately after the intervention was completed, all participants received via email a link to the same four questionnaires they had filled in prior to the intervention. The members of the control group received a premium membership that gave them access to all the contents in the app. All participants were encouraged to continue doing practices they found most beneficial. Two months after the completion of the intervention all participants were sent via email a link to the same four questionnaires to be filled in for the third time.

#### Intervention

The mindfulness-based intervention was carried out using a smart phone app developed by Welzen (Atlanta, Georgia, USA) specifically for this study. The 7-day mindfulness program was targeted to alleviate symptoms of psychological stress, anxiety and depression as well as increase mindfulness skills. The intervention group repeated the 7-day program four times by carrying out one practice each day. Thus, the intervention lasted for 28 days. Each guided mediation developed and performed by Lena Franklin lasted for approximately 10 min. The guided meditations trained the participants to recognize bodily signs of psychological stress, to focus attention to breathing, to enhance patience, to find balance between external requirements and the inner self, to calm down and relax via breathing, and to strengthen self-compassion via a loving-kindness meditation.

The control group listened to 2–5-min mini-lectures on mindfulness practice. There were 12 lectures the control group listened to twice 6 days a week, repeating the protocol every week for four weeks. Thus, the psychoeducation program also lasted for 28 days. The lectures provided information on mindfulness practice regarding stress (three lectures), anxiety (3 lectures), paying attention (two lectures), and social relations (three lectures).

### Measures

#### Perceived Stress

Perceived stress was measured by the Perceived Stress Scale (PSS; Cohen et al., 1983). We translated it into Finnish, after which we asked a professional translator to translate it back to English. The back-translation was then compared to the original questionnaire. As a result, a few small revisions were made on the Finnish translation. PSS is a 10-item questionnaire that assesses how often a respondent has felt or thought in a certain way during the last month. A 5-point scale is used: never, almost never, sometimes, fairly often, very often. Every item is credited 0–4 points, so the range of the total score is 0–40. Higher scores denote increased perceived stress. The test has good reliability and validity (Cohen et al., 1983). In the present sample, the reliability was also estimated to be good (Cronbach’s alpha = .82).

We also employed a single-item stress question developed by the Finnish Institute Occupational Health (Elo et al., 2003) and shown to be a reliable indicator of perceived stress. The English translation of this question reads as follows: “Stress refers to a situation where a person feels tense, restless, nervous or anxious, or she or he has difficulty sleeping, because bothering issues upsets the mind. Do you currently feel this kind of stress?”

#### Anxiety

Anxiety was measured by the Generalized Anxiety Disorder (GAD-7) questionnaire (Spitzer et al., 2006) translated into Finnish by the Finnish Institute Occupational Health (http://www.thl.fi/toimia/tietokanta/mittariversio/109/). The respondent answers to seven questions concerning her/his feelings of worry, anxiousness, irritability, nervousness, restlesness, and fear during the last two weeks by using a 4-point scale: not at all sure, several days, over half the days, nearly every day. Each item is credited 0–3 points so that the range of the summed score is 0–21, bigger numbers denoting increased anxiety. The validity and reliability of the scale has been estimated to be satisfactory (Spitzer et al., 2006). In the present data set, its reliability was good (α = .82).

#### Depression

Symptoms of depression were measured by the Beck Depression Inventory (BDI-21; Beck et al., 1988) translated into Finnish by Aalto (2009). The respondent answers 21 items inquiring the person’s feelings and life situation during the past week. Each item receives a score from 0 to 3; thus, the summed score ranges from 0 to 63. The validity and reliability of the inventory has been estimated to be satisfactory (Beck et al., 1988). In the present data set, its reliability was assessed to be good (α = .85).

#### Mindfulness

Mindfulness skills were measured a by an abbreviated (24 items) version (FFMQ-SF; Bohlmeijer et al., 2011) of the Five Facet Mindfulness Questionnaire (FFMQ; Baer et al., 2008) translated into Finnish by Rantonen (2014). A 5-point Likert scale is used to answer the questions with the following response alternatives: never or very rarely true, rarely true, sometimes true, often true, very often or always true. The five facets of mindfulness measured by the questionnaire are observing, describing, acting with awareness, non-judging of inner experience, and non-reactivity to inner experience. The sum score varies from 5 to 120. Both versions of the questionnaire are estimated to be valid and reliable indicators of mindfulness (Baer et al., 2008; Bohlmeijer et al., 2011). In the present study, the reliability of FFMQ-SF was good (α = .85).

#### Meditation Practice Duration (Minutes)

A meditation practice minute counter was developed by the app firm and embedded within the app. When the participant practiced mindfulness using the app, his/her practice minutes were recorded and stored.

#### Cortisol Levels

For a small subset of participants (*n* = 30) in the intervention group, cortisol levels from saliva were collected and analyzed pre- and post-intervention. The sample was taken randomly from the intervention group and limited to 30 due to financial reasons. Three daily samples (after waking up, 30 min after walking up, and right before going to bed) were collected in three consecutive days (two working days and one leisure day). Thus, 18 samples were collected from each participant. They were instructed not to eat or drink 15–30 min prior to taking the sample and wash their mouth with water after eating. Moreover, detailed instructions were provided about how to take the sample and place it in the test tube. A return envelope was also provided for mailing the test tubes to the Finnish Institute of Occupational Health, where the samples were analyzed. One participant failed to return samples and thus, cortisol data was available from 29 participants.

### Statistical Analyses

The data were analyzed with linear mixed models using the *lme4* (1.1–21; Bates et al., 2015) and cumulative link mixed models using the *ordinal* (version 2019.12–10; Christensen, 2019) packages for *R* statistical software (version 3.6.1; R Core team, 2019). All dependent variables were analyzed with linear mixed models, except for the single item stress question, which was analyzed with a cumulative link mixed model. Separate models for each dependent variable were conducted, and they were of the form DV ~ Time * Group + (1|Participant), in which Time of testing (pre and post) and Group (control vs. treatment) were entered as sum coded fixed effects. Random intercept for participants was included in the random part of the model. Observations exceeding 3 SDs were excluded as outliers from the analyses reported (for the PSS score, 1 participant, and for the GAD and BDI scores, 3 participants were dropped). Analyses were also conducted on all data, and even though this did not change the overall pattern of results, the effects for PSS and BDI scores failed to reach significance when the outliers were included in the analyses. Follow-up comparisons for interactions were conducted with the *emmeans* package (version 1.4.5; Lenth et al., 2020) by computing contrasts between control and treatment groups. Cohen’s d for the difference between pre- and post-test was calculated using a pooled estimate of the pre- and post-test variance.

Cortisol measurements were collected from a subsample of the treatment group (*N* = 29), and changes between the pre- and post-test in cortisol were examined with a model DV ~ Time + (1|Participant), in which Time of testing (pre and post) was entered as a sum coded fixed effect. Note that there were 9 cortisol measurements at both pre and post-test phases.

As specified in the preregistration, exploratory analyses were conducted to examine whether treatment effects depended on the level of mindfulness after treatment. The post-test mindfulness score was centered and entered into the models as a fixed effect, including its interaction effects with Group and Time. We also explored differences in treatment effects between students, faculty and staff by entering participant group as a fixed effect in the models (bachelor’s degree students as the baseline group), including its interaction effects with Group and Time. In addition to these preregistered exploratory analyses, we also examined whether treatment effects observed in the treatment group were associated with practice time (i.e., minutes of app use). In these analyses, practice time was centered and entered in the models as a fixed effect, including its interaction terms with Time. As the control group had access to the app after the post-test, we also examined practice effects during the whole study period (from the pretest to the follow-up) for all participants.

## Results

### Perceived Stress

In the analysis of the PSS scores (see Table 1), there was a main effect of time, indicating that the scores decreased during the intervention period. Importantly, a Time*Group interaction indicates that the scores decreased more in the treatment (Cohen’s *d* = −.50) than in the control group (*d* = −.34). However, in the follow-up comparisons the difference between control and treatment groups at post-test failed to reach statistical significance, estimate = .83, *SE* = .50, *t* = 1.66, *p* = .097.

**Table 1:** Model estimates for the PSS scores

	PSS		
Predictor	*b*	*CI*	*t*
Intercept	16.51	16.10–16.91	80.01
Time	1.09	0.86–1.31	9.40
Group	0.19	−0.22 – 0.59	0.91
Time: Group	−0.23	−0.45 – −0.00	−1.96
Random Effects			
σ^2^	11.46		
τ_00 user_	16.37		
N _user_	560		
Observations	965		

The analysis of the single item stress question (see Table 2) showed that the score decreased during the intervention period (*d* = −.43), and that overall, the treatment group had lower stress scores. However, there was no indication of an interaction.

**Table 2:** Model estimates for the single item stress question

	Single item stress question		
Predictor	*OR*	*CI*	*Z*
Time	1.81	1.56–2.09	8.02
Group	1.24	1.00–1.52	2.00
Time: Group	0.92	0.80–1.05	−1.28
Random Effects			
σ^2^	3.29		
τ_00 user_	3.61		
N _user_	561		
Observations	967		

### Anxiety

The analysis of the GAD scores (see Table 3) showed a main effect of time, indicating that the scores decreased during the intervention period (*d* = −.40). However, there was no indication of an interaction involving Group.

**Table 3:** Model estimates for the anxiety scores

	GAD		
Predictor	*b*	*CI*	*t*
Intercept	12.79	12.53–13.06	93.16
Time	0.66	0.51–0.82	8.38
Group	0.08	−0.19 – 0.34	0.55
Time: Group	−0.12	−0.27 – 0.04	−1.47
Random Effects			
σ^2^	5.36		
τ_00 user_	7.03		
N _user_	558		
Observations	958		

### Depression

In the analysis of the BDI scores (see Table 4) there was a main effect of time, indicating that the scores decreased during the intervention period. Moreover, a Time * Group interaction suggests that the scores decreased more in the treatment (*d* = −.45) than in the control group (*d* = −.29). In the follow-up comparisons, the difference between control and treatment groups at post-test failed to reach significance, estimate = .88, *SE* = .56, *t* = 1.59, *p* = .11.

**Table 4:** Model estimates for depression scores

	BDI		
Predictor	Estimate	CI	t
Intercept	8.09	7.63–8.55	34.67
Time	0.96	0.72–1.21	7.77
Group	0.20	−0.26 – 0.65	0.84
Time: Group	−0.25	−0.49 – −0.00	−1.99
Random Effects			
σ^2^	12.94		
τ_00 user_	21.85		
N _user_	558		
Observations	956		

### Mindfulness

The analysis of the FFMQ scores (see Table 5) showed an overall increase in the scores during the intervention period (*d* = .24), but no indication of an interaction.

**Table 5:** Model estimates for the mindfulness scores

	FFMQ		
Predictor	Estimate	CI	t
Intercept	83.12	82.21–84.03	179.87
Time	−1.22	−1.63 – −0.80	−5.78
Group	−0.29	−1.20 – 0.61	−0.63
Time: Group	0.36	−0.05 – 0.77	1.71
Random Effects			
σ^2^	37.21		
τ_00 user_	95.00		
N _user_	561		
Observations	966		

### Cortisol Levels

Cortisol data was collected from 29 participants. Of these, four did not provide samples in the post-test phase. There was no indication of changes in the cortisol levels in the treatment group (*d* = .06; see Table 6).

**Table 6:** Model estimates for cortisol (nmol/l)

	Cortisol		
Predictor	Estimate	CI	t
Intercept	7.52	6.65–8.40	16.79
Time	−.16	−.74 – -.43	−.53
Random Effects			
σ^2^	40.11		
τ_00 user_	3.26		
N _user_	29		
Observations	470		

### Exploratory Analyses of the Relationship between Treatment Effects and Mindfulness Skills

The analyses with the post-test FFMQ score as a covariate in the models did not provide evidence for three-way interactions between time, treatment group and mindfulness after treatment. There were two-way interactions between time and FFMQ post-test score for PSS (*b* = .39, *SE* = .18, *t* = 3.28), single item stress question (*b* = .17, *SE* = .07, *z* = 2.30), and BDI (*b* = .41, *SE* = .13, *t* = 3.22), indicating that the decrease in these scores were greater for participants who reported higher mindfulness after the treatment period.

### Exploratory Analyses of the Treatment Effects in Different Participant Groups

There were no indications of three-way interactions between time, treatment group and participant group.

### Exploratory Analyses of the Relationship between Treatment Effects and App Use (Dose-Response Relationship)

The total app use in the intervention group ranged from 8.6 min to 678.7 min (*M* = 195.6 min). The participants assigned to the intervention group used the app from 1 to 71 times, with an average of 21.4 times, during the intervention. In the analyses with total time that the app was used during the intervention as one of the fixed effects, there was a Time * Practice time interaction in the FFMQ scores, *b* = −.74, *SE* = .36, *t* = −2.06, indicating that the more the app was used, the more the FFMQ scores increased from pre- to post-test in the intervention group. No indication of interaction effects was observed in the other measures.

The control group got access to the app after the intervention period, so we also analyzed whether the app use in both the control and the intervention groups was related to changes in the independent measures between the pre-test and follow-up. The total app use during the whole study period ranged from 2.5 min to 1985.7 min (*M* = 235.9 min). The participants used the app from 1 to 165 times, with an average of 24.8 times, during the whole study period. There were no indications of interaction effects involving practice time in any of the measures.

## Discussion

We set out to investigate whether a 4-week course on a mindfulness app would result in wellbeing benefits over and above those from an online mindfulness course without the active ingredient of actual meditation practice. We also assessed whether more time spent practicing on the one hand, and increases in mindfulness on the other hand, would result in additional benefit to participants. Our results indicate that practicing mindfulness with the help of a mindfulness app resulted in alleviated stress (though not when measured through the single-item stress question) and depression. The results for anxiety were also close to statistical significance. The effects are evident in comparison with an active control group, which is a gold standard in intervention research. Thus, the present study provides some evidence of the efficacy of using a mindfulness app to alleviate mental suffering. Contrary to our expectation, more time spent practicing did not predict further alleviation in stress, depression, or anxiety, despite the fact that the degree of perceived mindfulness increased as a function of practice time.

Our results regarding intervention effects are in line with the previous literature, which has indicated lessened anxiety and depression and improved wellbeing from practicing mindfulness using apps (e.g., Bostock et al., 2019; Clarke & Draper, 2020). Furthermore, our study provides further evidence for a beneficial intervention effect on stress, as reported for online mindfulness-based interventions in the meta-analysis of Spijkerman et al. (2016), which was not conclusively demonstrated for face-to-face MBIs (see the meta-analysis of Goyal et al., 2014). Finally, cortisol levels were not found to be decreased by the intervention. Yet, the small sample size likely played a role in burying any possible effect. As biological measures may become more readily available in the future, studies would do well to include them more as sensitive and objective outcome measures.

As the need for large-scale, cost-effective, low-risk interventions in mental health is large and growing, our study provides crucial evidence their usability at least in a university student and staff population like the one examined in the present study. The 2020 pandemic situation has created further anxiety and stress in the society and has also made, for the time being, face-to-face mindfulness-based interventions nearly impossible to administer with restrictions on people gathering in the same space. As evidence of their benefits accumulate, mindfulness apps are likely to increasingly meet the need of people searching for peace and calm amid stormy life circumstances.

Time spent practicing did not modify the intervention effects. This was likely due to there being relatively little variance in the practice times. The app offered a 10-min daily meditation practice for participants and there was no added incentive to practice more. As the mean daily practice duration was between 8 and 9 min, participants were quite diligent in their practice routines. It is encouraging that positive intervention effects were obtained with relatively small daily time investments. Yet, effects from additional practice would have needed more extensive individual variability in practice time. Finally, it may be noted that individual variability in the perceived mindfulness did not modify the observed intervention effects. This may not be very surprising given the fact that mindfulness measures are somewhat unreliable indicators for actual increases in mindfulness; thus, salutary effects may have been lost in noise (Goleman & Davidson, 2017).

### Limitations, Strengths, and Future Directions

Even though the study was uncommonly robust for the field (a large sample size combined with an active control group), some cautionary notes are in order. First, even though attrition rate was lower than has been reported for non-mindfulness mHealth interventions (31%; Melville et al., 2010), the study’s rate of 28.0% could still have been lower, perhaps with the help of gamification in app design, by adding incentives to remain in the study via a reward system, or by providing more encouraging feedback. Second, the sex balance in the sample was lopsided with more than five times more females than males. This is a common finding in volunteer-based mindfulness studies, as females appear to volunteer more often than males to mindfulness studies (e.g., Lahtinen & Salmivalli, 2020). Using a sample of volunteers is also a limitation, as there may be a reason to self-select, which leads to bias. The sample may then include an excessive number of people eager to meditate, or, on the other hand, participants with pronounced suffering in their lives. Third, the saliva sample subgroup in our study was too small to draw firm conclusions related to effects on stress-hormone levels. Moreover, due to financial reasons the cortisol group had no control group. Thus, these results should be treated with appropriate caution.

Fourth, the active control, though an unusually robust control condition for the field, could still have been designed to more closely resemble the treatment condition – it could have been an app otherwise identical to the main app, just without the meditation practices. The best studies in this regard have offered a “placebo” treatment as an active control condition via the same modality as the treatment condition, e.g. the Health Enhancement Program used in many face-to-face MBI studies (Eisendrath et al., 2014; Goleman & Davidson, 2017; MacCoon et al., 2012). Fifth, the use of self-report measures for main outcomes may be considered a limitation, though this is how depression and anxiety are commonly measured.

Notable strengths in the study are its large sample size, its robust active control condition, its implementation of strict estimates of actual meditation practice minutes and its preregistration. The methodology in much of mindfulness research has been notoriously shoddy (Coronado-Montoya et al., 2016; Goleman & Davidson, 2017; Van Dam et al., 2018). Thus, high-quality meta-analyses typically can only include <5% of all available studies due to lapses in methodological rigor (Goyal et al., 2014; Kuyken et al., 2015). As the most popular mindfulness apps now have users in the millions, the future of mindfulness practice is likely to increasingly involve the use of apps. High-quality studies evaluating their efficacy are thus likely to be in high demand and the field should take up the gauntlet.
